# Oleacein Attenuates the Pathogenesis of Experimental Autoimmune Encephalomyelitis through Both Antioxidant and Anti-Inflammatory Effects

**DOI:** 10.3390/antiox9111161

**Published:** 2020-11-21

**Authors:** Beatriz Gutiérrez-Miranda, Isabel Gallardo, Eleni Melliou, Isabel Cabero, Yolanda Álvarez, Prokopios Magiatis, Marita Hernández, María Luisa Nieto

**Affiliations:** 1 Instituto de Biología y Genética Molecular (IBGM-CSIC/UVa), 47001 Valladolid, Spain; bgutimiranda@gmail.com (B.G.-M.); isa_gallard@hotmail.com (I.G.); netmabel@hotmail.com (I.C.); yolipi78@hotmail.com (Y.Á.); maritahg@ibgm.uva.es (M.H.); 2 Laboratory of Pharmacognosy and Natural Products Chemistry, Department of Pharmacy, National and Kapodistrian University of Athens, Panepistimiopolis Zografou, 15771 Athens, Greece; emelliou@pharm.uoa.gr (E.M.); magiatis@pharm.uoa.gr (P.M.); 3 Departamento de Bioquímica y Biología Molecular, Facultad de Medicina, Universidad de Valladolid, 47001 Valladolid, Spain

**Keywords:** EAE, multiple sclerosis, inflammation, cytokines, oleacein, oxidative stress, secoiridoid, polyphenol, EVOO

## Abstract

Oxidative stress and proinflammatory cytokines are factors affecting multiple sclerosis (MS) disease progression. Oleacein (OLE), an olive secoiridoid, possesses powerful antioxidant and anti-inflammatory activities, which suggests its potential application to treat neuroinflammatory disorders. Herein, we investigated the impact of OLE on the main clinic-pathological features of experimental autoimmune encephalomyelitis (EAE), an animal model for MS, including paralysis, demyelination, central nervous system (CNS) inflammation/oxidative stress and blood-brain barrier (BBB) breakdown. Methods: Mice were immunized with the myelin oligodendrocyte glycoprotein peptide, MOG_35-55,_ to induce EAE, and OLE was administrated from immunization day. Serum, optic nerve, spinal cord and cerebellum were collected to evaluate immunomodulatory activities at a systemic level, as well as within the CNS. Additionally, BV2 microglia and the retinal ganglion cell line RGC-5 were used to confirm the direct effect of OLE on CNS-resident cells. Results: We show that OLE treatment effectively reduced clinical score and histological signs typical of EAE. Histological evaluation confirmed a decrease in leukocyte infiltration, demyelination, BBB disruption and superoxide anion accumulation in CNS tissues of OLE-treated EAE mice compared to untreated ones. OLE significantly decreased expression of proinflammatory cytokines (IL-13, TNFα, GM-CSF, MCP-1 and IL-1β), while it increased the anti-inflammatory cytokine IL-10. Serum levels of anti-MOG_35-55_ antibodies were also lower in OLE-treated EAE mice. Further, OLE significantly diminished the presence of oxidative system parameters, while upregulated the ROS disruptor, Sestrin-3. Mechanistically, OLE prevented NLRP3 expression, phosphorylation of p65-NF-κB and reduced the synthesis of proinflammatory mediators induced by relevant inflammatory stimuli in BV2 cells. OLE did not affect viability or the phagocytic capabilities of BV2 microglia. In addition, apoptosis of RGC-5 induced by oxidative stressors was also prevented by OLE. Conclusion: Altogether, our results show that the antioxidant and anti-inflammatory OLE has neuroprotective effects in the CNS of EAE mice, pointing out this natural product as a candidate to consider for research on MS treatments.

## 1. Introduction

Secoiridoids, a group of phytochemicals occurring wide-spread in nature, exhibit a broad range of biological and pharmacological activities, including antibacterial, anticancer, anticoagulant, antifungal, antioxidant, antiprotozoal and hepatoprotective activities [1,2]. The secoiridoid derivative of oleuropein, oleacein, is the dialdehydic form of decarboxymethyl elenolic acid esterified with 3, 4-(dihydroxyphenyl)ethanol (3,4-DHPEA-EDA). Oleacein (OLE) is one of the most abundant phenolic secoiridoids present in extra-virgin olive oil (EVOO), the main fat source in the Mediterranean diet, and has been proposed by many studies as a protective factor in a wide range of diseases, some of them with immunological and/or inflammatory profiles, where oxidation plays a key role [2,3,4].

Multiple Sclerosis (MS) shares those characteristics; being a chronic inflammatory and demyelinating disease of the central nervous system (CNS) that can affect spinal cord, brain and optic nerves [5]. The inflammatory process involves breakdown of the blood-brain barrier (BBB), as well as activation and recruitment of lymphocytes, microglia, and macrophages into the CNS to lesion sites. The secretion and accumulation of cytotoxic factors such as proinflammatory cytokines, chemokines and reactive oxygen species (ROS) contribute to myelin loss, resulting in oligodendrocyte destruction and axonal injury [6,7,8]. The trigger to the disease is still unknown and, as yet, it has to be definitively demonstrated that the immune response is the cause, and not a consequence, of some as yet unidentified process.

Numerous studies on MS have focused on the mechanisms that are hypothesized to underlie the CNS inflammation. Accordingly, MS therapies rely on manipulation of the immune system to modify its activity, easing inflammation. However, although disease-modifying therapies (DMT) are important treatment options, they carry a number of limitations, such as significant adverse side effects, and the relative and long-term benefits remain unclear. Therefore, introduction of novel treatments, that can act either alone or synergistically with existing therapies, would be highly beneficial for the treatment of MS.

The most commonly used autoimmune model in mice, experimental autoimmune encephalomyelitis (EAE), shares many similarities with MS and has been the main approach used for studying disease mechanisms and testing potential therapies for MS [9,10]. EAE is a CD4^+^ T cell-mediated autoimmune disease in which CNS inflammation is induced by active immunization with myelin-derived proteins or peptides that induce myelin-specific T cells. These autoreactive T cells initiate an inflammatory cascade through recruitment of monocytes and neutrophils to the CNS, which results in demyelination of axonal tracks, impaired axonal conduction in the CNS, and progressive hind-limb paralysis.

Recently, it has been shown that oleacein presents anti-inflammatory effects in human adipocytes challenged with TNFα, a prototypic inflammatory stimulus, which could aid to explain the cardiometabolic benefit of EVOO consumption [11]. However, at present, there are no data on the effects of oleacein on neurodegenerative disorders in general, MS in particular. In our study, we found that oleacein can ameliorate the clinical signs of EAE by modulating some of the immune–inflammatory and oxidative responses implicated in the etiology of this disease.

## 2. Material and Methods

### 2.1. Oleacein Isolation

Oleacein was isolated from appropriately selected olive oil after screening by NMR of 500 samples collected from Greece the harvest season 2015–2016. The selected oil contained the highest amount of oleacein among the studied samples. Olive oil from *Olea europaea* cv Koroneiki (500 g) from northern Peloponnese, Greece, was mixed with cyclohexane (2 L) and extracted with acetonitrile (2.5 L). The acetonitrile phase was collected and evaporated using a rotary evaporator under reduced pressure. The residue was submitted to column chromatography using Si gel 60 Merck (40–63 μm) as stationary phase and mobile phase, as shown in Table 1:

### 2.2. Disease Induction and Treatment

Female 8–10-week-old C57BL/J6 mice (from Charles River Laboratories, Barcelona, Spain) were housed in the animal care facility at the Medical School of the University of Valladolid and provided with food and water ad libitum. All animal care and experimental protocols were reviewed and approved by the Animal Ethics Committee of the University of Valladolid and complied with the European Communities directive 86/609/ECC and Spanish legislation (BOE 252/34367-91, 2005) regulating animal research.

#### 2.2.1. Induction of EAE

EAE was induced by subcutaneous immunization with 100 µg of myelin oligodendrocyte glycoprotein (MOG)_35–55_ peptide (MEVGWYRSPFSRVVHLYRNGK; from Dr F. Barahona, CBM, Madrid, Spain) emulsified in complete Freund’s adjuvant containing 0.4 mg *Mycobacterium tuberculosis* (H37Ra; Difco, Detroit, MI, USA) on day 0. Additionally, mice received 300 ng of Pertussis toxin injected intraperitoneally (i.p.) on days 0 and 2. Animals were monitored blindly and daily by 2 independent observers and neurological signs were assessed on a scale of 0 to 5, with 0.5 points for intermediate clinical findings: grade 0, no abnormality; grade 0.5, partial loss/reduced tail tone, assessed by inability to curl the distal end of the tail; grade 1, tail atony; grade 1.5, slightly/moderately clumsy gait, impaired righting ability or combination; grade 2, hind limb weakness; grade 2.5, partial hind limb paralysis; grade 3, complete hind limb paralysis; grade 3.5, complete hind limb paralysis and fore limb weakness; grade 4, tetraplegic; grade 5, moribund state or death. Scores from 2 investigators, both unaware of the treatments, were averaged. Data were plotted as daily mean clinical score for all animals in a particular treatment group. Scores of asymptomatic mice (score = 0) were included in the calculation of the daily mean clinical score for each group. The onset of EAE was defined as the first day an animal exhibited a clinical score ≥1.

#### 2.2.2. Oleacein Treatment

EAE mice received daily i.p. injection with vehicle control (Dimethyl sulfoxide (DMSO)/saline, *n* = 11) or 10 mg/kg of OLE (*n* = 11) starting from immunization day until the end of the experiment: approximately 22–24 days postimmunization, when EAE-mice showed hind limb paralysis. Control mice (without EAE induction) were also injected daily with OLE (*n* = 9) or vehicle control (*n* = 9) for an equivalent time frame. In total, 2 independent experiments with similar results were performed. Mice diet does not contain olive oil or any olive derived product which would be the source of oleacein and could skew the results.

OLE was dissolved in normal saline containing 5% DMSO. Stock solution of oleacein was prepared in DMSO at a concentration of 100 mg/mL and kept at 4 °C.

### 2.3. Histological Studies

CNS tissues (cerebellum, spinal cord and optic nerve) from mice from the different experimental groups were dissected on day 23 or 24 after immunization (*n* = 5/group). Tissues were fixed in 4% paraformaldehyde and embedded in paraffin. Paraffin-embedded tissues were cut on a microtome (5 μm thickness) and stained with hematoxylin and eosin (H&E) or with Luxol Fast Blue (LFB) for analysis of inflammation or demyelination, respectively. A qualitative evaluation was performed, in a blinded fashion, in each specimen to control the changes that occur along the treatment. Histopathological examination was performed with a Nikon Eclipse 90i connected to a digital photomicrographic camera DXM1200C (both from Nikon Instruments Inc., Amstelveen, The Netherlands).

### 2.4. BBB Permeability Measurement by IgG Extravasation

To detect BBB disruption, histological analysis of IgG extravasation was performed in the brain of control or EAE mice (untreated and treated with OLE) at 23 days after immunization. Free-floating 30 μm coronal sections from the brain were immunostained with FITC rat anti-mouse IgG (Vector, Burlingame, CA, USA). Histopathological examination was performed with a Nikon Eclipse 90i connected to a digital photomicrographic camera DXM1200C (both from Nikon Instruments Inc., Amstelveen, The Netherlands). The intensity of fluorescence signals was quantified using ImageJ software (NIH, Bethesda, MD, USA). A single researcher unaware of the experimental groups performed the analysis.

### 2.5. Inflammatory Markers and MOG_35–55_-specific IgG Quantitation

Serum was collected from animals on day 24 after immunization, aliquoted and stored at −80 °C. Level of antibodies directed against MOG_35–55_ was determined using the enzyme-linked immunoassay (ELISA) technique. Then, 96-well polystyrene microtiter plates were coated with 1 μg/well of MOG_35–55_ peptide by overnight incubation in PBS at 4 °C. After blocking with 5% BSA for 2h, the wells were incubated in duplicate with the serum samples diluted 1:60 in PBS for 2 h at room temperature. After washing, an HRP-conjugated goat antimouse IgG1 (1:1000) from Serotec (Sigma-Aldrich, St Louis, MO, USA) was added for 90 min. After another washing, adding the substrate, and arresting the reaction with 0.1N HCl, absorbance was read at 450 nm. The results were expressed as mean optical density at 450 nm.

For inflammatory markers quantification—TNFα, IL-1β and IL-10 and monocyte chemoattractant protein-1 (MCP-1)—serum and spinal cord tissue were analyzed by commercial ELISA kits according to the manufacturer’s protocols (eBioscience, San Diego, CA, USA).

### 2.6. Analysis of Oxidative Stress Markers in CNS Tissues

#### 2.6.1. CNS Tissues Preparations for Biochemical and Histopathological Changes

Parts of the freshly collected spinal cord were weighed, homogenized in PBS, pH 7.5, (1:10, w:v) and centrifuged at 1500× *g* for 10 min at 4 °C. The supernatant was recovered, set on ice and measured for oxidative products analysis.

Cerebellum, spinal cord and optic nerve from mice from the different experimental groups were collected, and frozen immediately in Optimal Cutting Temperature (OCT) compound (Tissue-Tek Tissue-Tek, Sakura Finetek USA, Inc. Torrance, CA, USA). Tissues were sectioned at 16 μm on a cryostat (HM 550 Serie, MICROM International GmbH, Walldorf, Germany).

#### 2.6.2. Detection of Superoxide Anion Production (O_2_^−^)

The oxidative fluorescent dye dihydro-ethidium (DHE) was used to evaluate production of O_2_^−^. DHE oxidation yields ethidium fluorescent compounds. Briefly, 16 μm thick sections of cerebellum, spinal cord and optic nerve were equilibrated in Krebs-HEPES buffer (NaCl 130 mM, KCl 5.6 mM, CaCl_2_ 2 mM, MgCl_2_ 0.24 mM, HEPES 8.3 mM, glucose 11 mM, pH 7.4). Fresh made buffer containing 5 µM of DHE was then added and incubated for 30 min at 37 °C. After that, sections were viewed by fluorescence microscope (Nikon 165 TE2000, Shinagawa-ku, Tokyo, Japan) under a 4×, 10× or 20× objective (40×, 100× and 200× final magnification, respectively). At least 5 images of each sample were captured for analysis using fixed exposure time for all groups. The intensity of fluorescence signals was quantified using ImageJ software (NIH, Bethesda, MD, USA). A single researcher, unaware of the experimental groups, performed the analysis.

#### 2.6.3. Analysis of Ferric Reducing Antioxidant Power (FRAP)

The FRAP assay was used to measure the total antioxidant activity in the serum and spinal cord tissue using the method described by Benzie and Strain [12,13]. FRAP values were calculated according to the calibration curve for FeSO_4_·7H_2_O and expressed as µM of Fe^2+^ equivalents.

#### 2.6.4. Determination of Malondialdehyde (MDA)

Lipid peroxidation level was measured spectrophotometrically by estimation of MDA concentration based on the reaction with thiobarbituric acid [14,15]. Briefly, spinal cord supernatants/serum were added to a reaction mixture consisting of 0.373% thiobarbituric acid, 15% trichloroacetic acid and 0.015% butylated hydroxytoluene (BHT). The mixture was then heated at 95 °C for 40 min, and was cleared by centrifugation at 3.800 rpm for 10 min. The absorbance was examined at 532 nm using a 96-well plate in a spectrophotometer (VERSAmax Microplate Reader. Molecular Devices LLC, San Jose, CA, USA).

#### 2.6.5. Determination of Advanced Oxidation Protein Products (AOPP)

Spinal cord supernatants/serum (20 µL) from each mouse were diluted into 100 µL in PBS, followed by addition of 10 µL of 1.16 M KI, and 20 µL absolute acetic acid. The absorbance of the reaction mixture was immediately read at 340 nm on the VERSAmax microplate reader against a blank containing 100 µL PBS, 20 µL acetic acid, and 10 µL KI solution. AOPP were calibrated with a chloramine-T solution (0–100 µM) that absorbs at 340 nm in the presence of 10 µL of 1.16 M potassium iodide. AOPP concentrations were expressed as µM chloramine-T equivalents.

### 2.7. Ex Vivo Lymphoid Cell Culture and Analysis of Proliferation

To prepare single cell suspension, spleens were collected on day 24. Then, they were pressed through a wire mesh and washed with ice-cold PBS. Red blood cells (RBC) were lysed by 5 min incubation in RBC lysis buffer from eBioscience (San Diego, CA, USA). RBC-depleted splenocytes were cultured in triplicate in 96-well plates at 0.5 × 10^5^ cells/well. The cultures were stimulated with 100 µg/mL MOG_35–55_ in presence or absence of different doses of OLE. After 72 h, cell proliferation was analyzed using the Promega kit, Cell Titer 96RAqueous One Solution Cell Proliferation Assay (Promega Corporation, Madison, WI, USA), according to the manufacturer’s recommendations. Formazan product formation was determined by measuring the absorbance at 490 nm, as an assessment of the number of metabolically active cells. Cell proliferation was expressed as a percentage relative to the untreated control cells.

### 2.8. In Vitro Studies

#### 2.8.1. Cell Culture

The immortalized microglia cell line BV2, that exhibits phenotypic and functional properties comparable with those of primary microglia and hippocampal neurons, was a gift from Prof J. Bethea, Miller School of Medicine, Miami, FL, USA [16]. The retinal ganglion cell line (RGC-5), an established model of oxidative stress injury in vitro to mimic optic nerve injury and evaluate neuroprotective factors, was a gift from Dr. P. Boya, Centro de Investigaciones Biológicas Margarita Salas, CIB-CSIC, Madrid, Spain [17].

Both cell lines were cultured in DMEM containing 10% Fetal Bovine Serum (FBS), 25 mM glucose, 100 U/mL penicillin, and 100 µg/mL streptomycin, and were incubated under 5% CO_2_ at 37 °C. Oleacein was dissolved in DMSO at 50 mM and then in cell culture medium to a final concentration of 10 μM and 20 μM. The final concentration of DMSO did not exceed 0.04%, nor did it interfere with the performed assays.

#### 2.8.2. Viability Assay

Cell viability was evaluated by using the Promega kit (Madison, WI, USA), Cell Titer 96^®^ Aqueous One Solution Cell Proliferation Assay, according to the manufacturer’s recommendations. Briefly, BV2 cells were seeded in 96-well plates and serum starved for 24 h. Then, cells were incubated with or without 20% FBS, in the presence or absence of different doses of OLE. After 24 h of incubation, formazan product formation was assayed by recording the absorbance at 490 nm in a 96-well plate reader (OD value). Formazan is measured as an assessment of the number of metabolically active cells and expressed in percentages relative to FBS-stimulated cells. Cell viability was assessed by Trypan blue exclusion.

#### 2.8.3. Apoptosis Assay

RGC-5 cells were stimulated for 24 h in serum-free media with 200 µM of tert-butyl hydroperoxide (t-BOOH), in the presence or absence of different doses of OLE. Then, cells were resuspended in 100 µL binding buffer (10 mM HEPES, pH 7.4, 150 mM NaCl, 2.5 mM CaCl_2_, 1 mM MgCl_2_, 4% BSA), and incubated for 15 min with 2.5 ng/mL Annexin V-FITC. Subsequently, 400 µL of binding buffer was added and the cells were detected and quantified using a Flow Cytometer (Gallios™; Beckman Coulter, Fullerton, CA, USA).

#### 2.8.4. Western Blotting

BV2 cells were preincubated for 30 min with the indicated doses of OLE and stimulated with 0.1 µg/mL of LPS for 4 or 24 h. Then, cells were harvested in Laemmli SDS sample buffer. Protein extracts were separated by SDS-PAGE and transferred to polyvinylidene difluoride membranes. Membranes were blocked with 5% bovine serum albumin in Tris-buffered saline with 0.1 % Tween 20 ® (BSA-TBST) at room temperature and then incubated for 18 h at 4 °C with the indicated antibodies including p-p65-NFκB (Cell Signaling Technology, Danvers, MA, USA), NLRP3 (R&D Systems, Minneapolis, MN, USA), COX-2 (sc-1745, Santa Cruz Biotech, Santa Cruz, CA, USA), actin (A5441, Sigma-Aldrich, St Louis, MO,USA) and iNOS (BD Biosciences, Lexington, KY, USA). After washing with TBST buffer, a 1:2000 (*v*/*v*) dilution of horseradish peroxidase-labelled IgG was added at room temperature for 1 h. The blots were developed using enhanced chemiluminescence.

#### 2.8.5. Cytokines Analysis

Supernatants of BV2 cells stimulated with 0.1 µg/mL of LPS for 24 h in the presence of different doses of OLE were used to quantify TNFα and IL-1β production with specific ELISA kits according to the manufacturer’s protocol (Invitrogen; Carlsbad, CA, USA). The assays were performed in triplicate.

#### 2.8.6. Phagocytosis Assays

BV2 cells were stimulated for 24 h in serum-free media with 0.1 µg/mL of LPS, in the presence or absence of different doses of OLE. Then cells were exposed to 0.1 mg/mL of FITC-labelled dextran (MW 40 000) for 2 h. Noninternalized particles were removed by vigorous washing with cold PBS (pH 7.4) prior to measuring fluorescence at 480 nm excitation and 520 nm emission on a Flow Cytometer (Gallios™; Beckman Coulter, Fullerton, CA, USA). Phagocytosis was expressed as the percentage of BV-2 cells that had engulfed FITC-labelled dextran (FL1 fluorescence positive cells) over the total number of BV-2 cells. Data were collected from at least four independent experiments. To visualize the internalized dextran, cells were also analyzed on a Leica TCS SP5X confocal microscope with a ×60 oil objective (Leica Microsystems S.L.U., Mannheim, Germany).

#### 2.8.7. Measurement of Intracellular ROS

ROS levels were measured with the probe dichlorodihydrofluorescein diacetate (DCFH-DA; Molecular Probes, Eugene, OR, USA). After reacting with intracellular ROS, DCFH produces the highly fluorescent compound 2′,7′-dichlorofluorescein (DCF). Briefly, cells were incubated with 10 μM DCFH-DA for 30 min at 37 °C in the dark. Then, cells were washed with PBS, pH 7.4 and stimulated in serum-free medium with or without 0.1 µg/mL of LPS for 24 h, in the presence or absence of different doses of OLE. The intensity of fluorescence was examined by flow cytometry (Gallios™; Beckman Coulter, Fullerton, CA, USA). The mean fluorescence intensity in 10,000 cells was recorded (480 nm excitation/530 nm emission).

#### 2.8.8. Measurement of Superoxide Anion (O_2_^−^) Production

The oxidative fluorescent dye DHE (Invitrogen, Carlsbad, CA, USA) was used to evaluate the production of ion superoxide (O_2_^−^). RGC-5 cells were incubated for 24 h with either vehicle or the indicated stimuli in the presence or absence of 20 µM of OLE. Cells were then incubated with 2 µM DHE for 30 min in a light-protected humidified chamber at 37 °C. Cells were analyzed under a 40× objective with a Nikon Eclipse 90i (Nikon Instruments, Inc. Melville, NY, USA). A total of 3 different assays were each performed in duplicated. Representative images are shown. The intensity of fluorescence signals was quantified using ImageJ software (NIH, Bethesda, MD, USA). A single researcher, unaware of the experimental groups, performed the analysis.

### 2.9. Statistical Analyses

Data analyses were performed using one-way ANOVA. Bonferroni test was utilized for post hoc comparisons among multiple groups where appropriate. Results described as mean ± standard error of the mean (SEM). *p* Values < 0.05 were considered statistically significant. Statistical analyses were performed using the GraphPad Prism Version 4 software (San Diego, CA, USA).

## 3. Results

### 3.1. Oleacein Treatment Attenuates EAE Development

**Figure 1:**
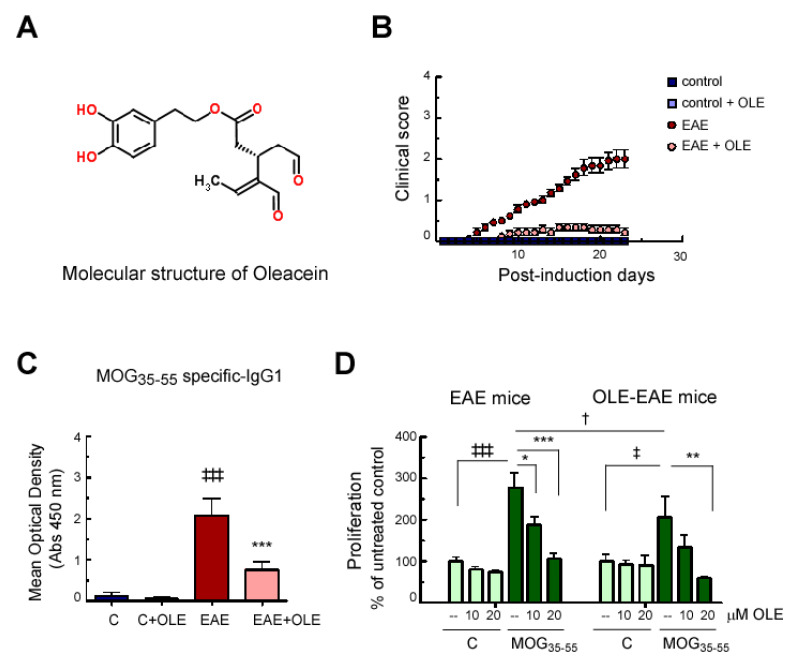
Oleacein (OLE) treatment protects mice from experimental autoimmune encephalomyelitis (EAE). (**A**) Chemical structure of Oleacein. (**B**) Clinical scores of EAE in mice treated with 10 mg/kg/day of OLE. The error bars represent the SEM for each point. Depicted are the combined results of two independent experiments (*n* = 9 in control and OLE-control groups; *n* = 11 in EAE and OLE-EAE groups) (**C**) Titers of MOG_35–55-_specific IgG1 in serum samples at 1/60 dilution. ^‡‡‡^ *p* < 0.001 vs. control and *** *p* < 0.001 vs. EAE. (**D**) Effect of OLE on splenocyte cell functions. In vitro, MOG_35–55_-specific proliferative response of splenic cells from EAE mice and OLE-treated EAE mice. T cell proliferation was measured in spleen lymphocyte cultures incubated for 72 h in the presence or absence of MOG_35–55_ and with or without the indicated dose of OLE. Spleen lymphocytes were obtained at day 24 after EAE induction. Histograms represent the mean ± SEM. Measures were performed in duplicated wells, *n* = 5 in each group, from two independent experiments. ^‡‡‡^ *p* < 0.001 and ^‡^ *p* < 0.05 vs. control; ^***^ *p* < 0.001, ** *p* < 0.01 and * *p* < 0.05 vs. MOG_35–55_ without OLE; ^†^ *p* < 0.05 vs. EAE mice.

We investigated the effect of OLE in EAE development. C57BL/6 female mice of 8 weeks-old were immunized with MOG_35–55_ as described in Material and Methods. OLE (10 mg/Kg body weight) was injected intraperitoneally to each mouse daily from immunization day. Clinical score was recorded daily from day 1 to day 23–24 postimmunization (Figure 1B). Control and OLE-treated control groups were not expected to exhibit clinical signs of disease. In contrast, the EAE group began to display symptoms around day 10. Analysis of EAE course showed a delay on the disease onset and a reduction of its severity by OLE treatment. First neurological symptoms (score 1) were observed at day 9 with mean day of onset 11.6 ± 2 in untreated EAE mice, while OLE-treated animals showed no clinical signs at that time and only minimal pathological abnormalities were developed. On day 24, while untreated EAE mice showed partial hind limb paralysis with an average clinical score of 2.5 ± 0.3, in the OLE-treated EAE group 9/11 mice showed inability to curl the distal end of the tail (score 0.5) and only 2/11 mice showed tail atony (score 1).

Next, we investigated whether this protective effect of OLE attenuating EAE clinical signs affected elements of the humoral and cell mediated immune response. Firstly, we evaluated whether OLE treatment impaired humoral immunity. As shown in Figure 1C, high levels of the MOG_35–55_-specific IgG1 subclass were found in serum of EAE mice, whereas serum from control and OLE-treated control animals showed an almost complete absence of MOG_35–55_ IgG1 antibody titers. In contrast, serum from OLE-treated EAE mice exhibited a significant reduction in this marker of the humoral immune reaction (64%).

After that, we studied whether OLE treatment compromised systemic immune responses. We evaluated the function of splenocytes collected from mice of the untreated-EAE and OLE-treated EAE groups, at day 24 post-immunization. When a lymphocyte encounters its antigen, it triggers the activation of the lymphocyte, and, among other responses, proliferation can be found. Spleen lymphocytes were assessed for antigen-specific proliferation after in vitro stimulation with MOG_35–55_ for 72 h in the presence or absence of different doses of OLE. As shown in Figure 1D, spleen cells from OLE-treated EAE mice respond with a lower proliferation rate to in vitro MOG_35–55_ challenge than those from untreated EAE mice. Moreover, the MOG_35–55_-specific proliferative response was significantly suppressed by the presence of OLE, in a dose-dependent manner.

### 3.2. Oleacein Protects from CNS Damage

**Figure 2:**
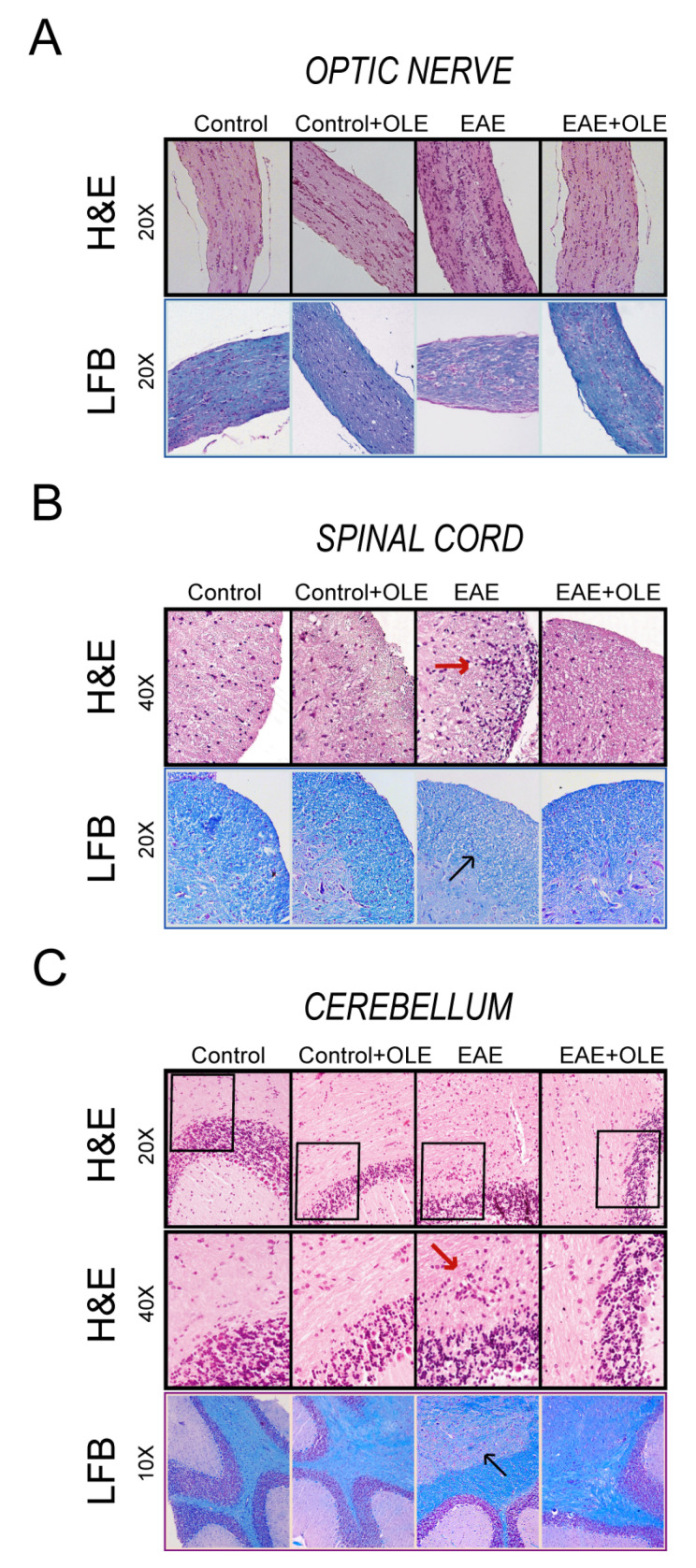
Oleacein treatment protects EAE mice from infiltration of inflammatory cells into central nervous system (CNS) and demyelination. Optic nerve (**A**), spinal cord (**B**) and cerebellum (**C**) from healthy-control and EAE mice treated with vehicle or OLE were removed on day 23 after induction and stained in with H&E in the upper panels or Luxol Fast Blue (LFB) in the lower ones. Representative sections are shown (*n* = 5/group, from two independent experiments.). Red arrows indicate infiltration and blue arrows indicated area of demyelination.

To determine whether OLE modulated the inflammatory response in EAE, we first assessed the occurrence of inflammatory cell infiltration in several CNS tissues collected at day 23–24 postimmunization (Figure 2). Examination of H&E stained sections of optic nerve, spinal cord and cerebellum showed the presence of cell infiltrates in all tissues from EAE mice. In contrast, the infiltrating cells in the CNS tissues from OLE-treated EAE mice were notably reduced, being comparable to those observed in tissues of untreated healthy-control mice.

Next, we addressed whether OLE protects from demyelination, by staining myelin in optic nerve, spinal cord and cerebellum with LFB. Untreated EAE mice showed a marked increase in demyelination compared to control mice. Unstained regions indicating destruction of the myelin sheath was observed in EAE mice tissues. Optic nerves of OLE-treated EAE mice showed increased LFB staining compared with optic nerves of untreated EAE mice, thus meaning suppression of the demyelination in optic nerves.

### 3.3. OLE Reduces Blood–Brain Barrier Disruption in EAE Mice

**Figure 3:**
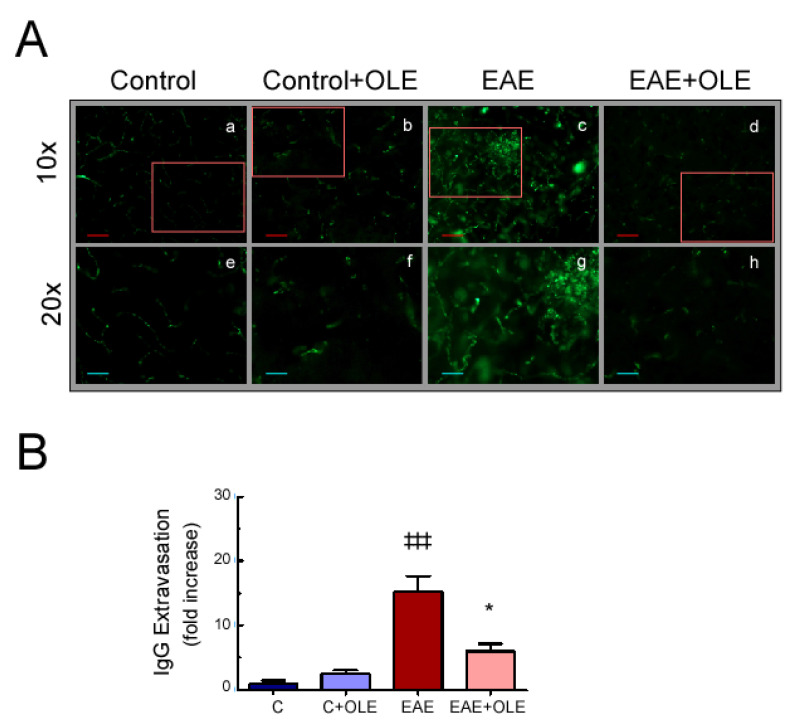
Oleacein treatment reduces blood–brain barrier disruption damage in EAE mice. (**A**) Fluorescence images represent immunohistochemical characterization of IgG extravasation on brain from control (**a**,**e**), OLE-treated control (**b**,**f**), EAE (**c**,**g**) and OLE-treated EAE (**d**,**h**) mice, at day 23 postimmunization. Representative sections from each group are shown (*n* = 5/group, from two independent experiments). (**B**) Quantification of IgG extravasation, ^‡‡‡^ *p* < 0.001 vs. control; and * *p* < 0.05 vs. EAE.

Blood-brain barrier (BBB) dysfunction is another relevant pathological hallmark of EAE pathogenesis. Consequently, we characterized the EAE-induced BBB damage in OLE treated and untreated mice by analysis of endogenous serum IgG leakage into the brain using immunohistochemistry. As shown in Figure 3A,B, brain from untreated EAE-mice revealed a marked increase in IgG extravasation (Figure 3(Ac,Ag)) compared to healthy control animals (Figure 3(Aa,Ae)). However, this enhanced permeability was prevented by the administration of OLE (Figure 3(Ad,Ah)). The treatment with OLE did not modify BBB integrity in control animals (Figure 3(Ab,Af)).

### 3.4. Oleacein Treatment Reduces the Production of Inflammatory Markers in EAE

**Figure 4:**
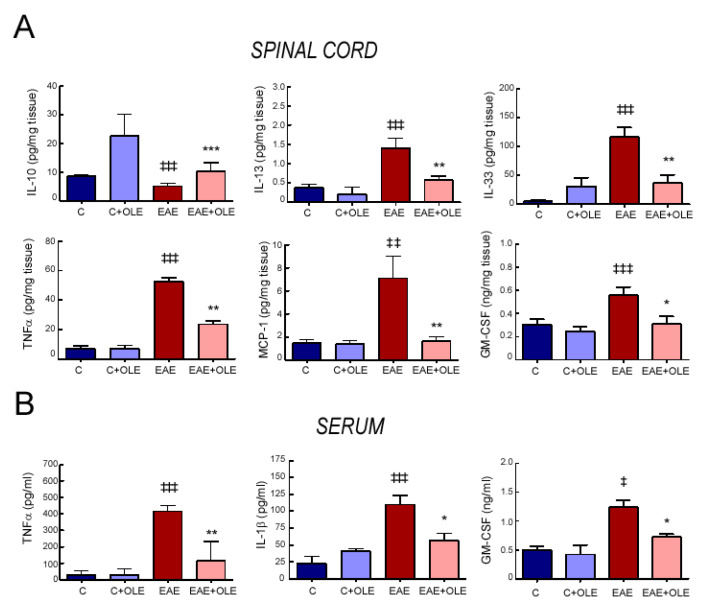
Oleacein treatment modulates production of inflammatory markers in EAE mice. Spinal cord (**A**) and serum (**B**) from control and EAE mice treated with vehicle or OLE were analyzed at day 23 post-immunization. Protein levels of GM-CSF, IL-13, IL-33, TNFα, MCP-1, IL-10 and IL-1β were quantified by commercial ELISA. Bar graphs represent the mean ± SEM of 5–8 animals, from two independent experiments.). ^‡‡‡^ *p* < 0.001, ^‡‡^ *p* < 0.01 and ^‡^ *p* < 0.05 vs. control; and *** *p* < 0.001, ** *p* < 0.01 and * *p* < 0.05 vs. EAE.

The ability of OLE to ameliorate the inflammatory response was further explored using spinal cord tissue, as a model of a CNS tissue specifically damaged in EAE, and the levels of inflammatory mediator were investigated (Figure 4). As expected, the expression of the inflammatory cytokine TNFα, the chemoattractant MCP-1, the immune modulator granulocyte-macrophage colony-stimulating factor (GM-CSF) as well as the immunoregulatory interleukins IL-13 and IL-33 were significantly increased in spinal cord from EAE mice, compared to the healthy control group. However, their overexpression was prevented in spinal cord tissue of EAE mice treated with OLE: levels were not significantly enhanced compared to those found in spinal cord of both treated- or untreated-control mice. Conversely, the levels of the anti-inflammatory cytokine IL-10 significantly decreased in EAE mice tissues in comparison with those in the control group and were augmented by OLE treatment. We also determined the circulating levels of TNFα and GM-CSF in serum samples, finding a similar pattern to those observed in the spinal cord. Furthermore, high levels of active IL-1β, a mediator connecting innate and adaptive immunity, were detected in serum from EAE mice and this increase was lower (~80%) in animals treated with OLE.

### 3.5. Oleacein Modulates Oxidative Stress in EAE Mice

It is well known that inflammation increases ROS levels leading to oxidative stress that mediates CNS damage in MS. To investigate whether prophylactic administration of OLE to EAE mice also resulted in reduced ROS accumulation, we used, as an initial gauge of ROS activity, the redox-sensitive fluorescent probe DHE that detects superoxide anion (O_2_^−^).

**Figure 5:**
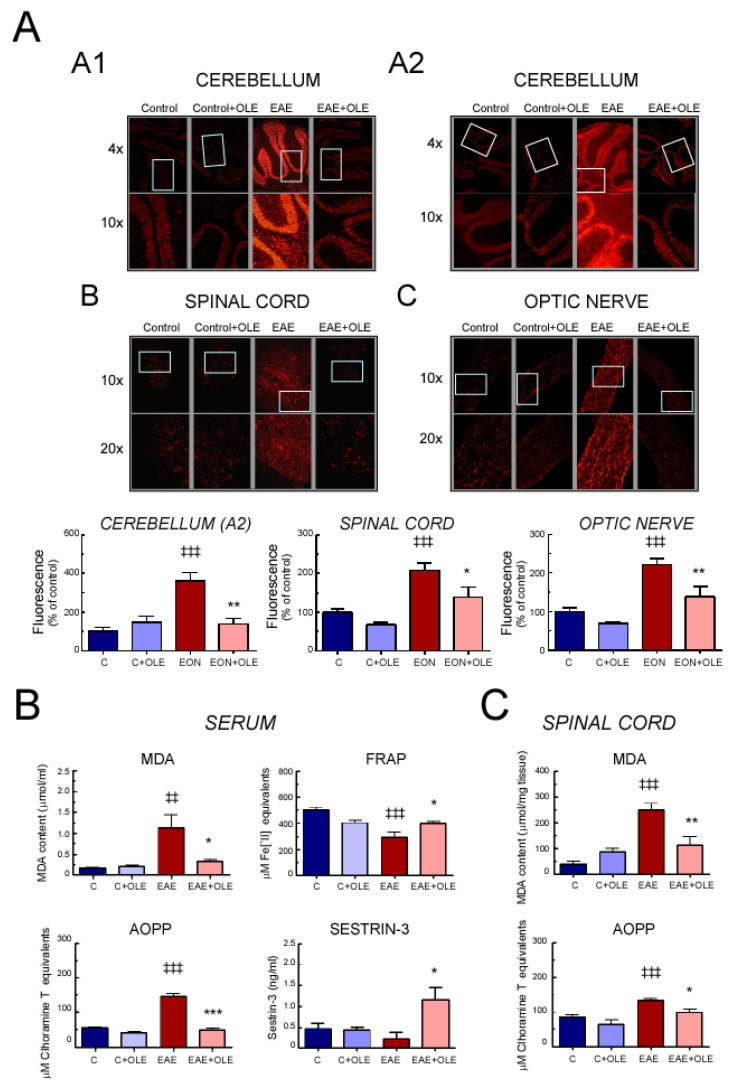
Oleacein treatment protects EAE mice from oxidative stress. Cerebellum, spinal cord and optic nerve from control and EAE mice treated with vehicle or OLE were analyzed at day 23 post-immunization. (**A**) Representative microphotographs of CNS tissues sections labeled with DHE, analyzed by fluorescence microscopy. Cerebellum: **AA1** and **AA2** panels represent the same sections visualized at different settings. Cerebellum: upper and lower panels represent a same section visualized with different magnification: 4× and 10× lens, respectively. Spinal cord (**AB**) and optic nerve (**AC**): upper and lower panels represent a same section visualized using a 10× or a 20× lens, respectively. Quantification graphs can be seen underneath the images. Levels of malondialdehyde (MDA), advanced oxidation protein products (AOPP) and ferric-reducing antioxidant power (FRAP) and sestrin-3 in serum (**B**), and MDA and AOPP levels in spinal cord (**C**) of the same animals. Bar graphs represent the mean ± SEM of 5–8 animals, from two independent experiments. ^‡‡‡^ *p* < 0.001 and ^‡‡^ *p* < 0.01 vs. control; and *** *p* < 0.001, ** *p* < 0.01 and * *p* < 0.05 vs. EAE.

Cryo-sections of cerebellum, spinal cord and optic nerve of mice from the different experimental groups were incubated with DHE stain and evaluated by fluorescence microscopy (Figure 5A). In healthy-control mice, DHE staining was basically undetectable in all the studied tissues. In contrast, tissues from EAE mice showed increased red fluorescence intensity throughout the CNS grey and white matter. However, OLE treatment inhibited EAE-induced ROS production: ethidium red fluorescence was extensively attenuated, being similar to that observed in tissues from healthy control mice.

Given that superoxide anion has also been implicated in both protein oxidation and lipid peroxidation, we assessed the levels of advanced oxidation protein products (AOPP) and malondialdehyde (MDA), as the end products of lipid peroxidation, in serum as well as in spinal cord, as a representative CNS tissue injured in EAE (Figure 5B,C). The EAE group showed significantly increased levels of both AOPP and MDA in serum and spinal cord tissue in comparison with the control group. Meanwhile, OLE treatment effectively prevented these increases. On the contrary, the serum antioxidant activity, evaluated by a ferric-reducing antioxidant potential (FRAP) assay, significantly decreased in EAE mice in comparison with that of control group and OLE treatment was able to ameliorate this drop, although it was unable to normalize it. Moreover, serum levels of the ROS disruptor Sestrin-3 were slightly lower in EAE mice compared to serum from control mice. The mere administration of OLE was able to remarkably increase its levels in serum from EAE mice. In spinal cord we found a similar pattern for FRAP and Sestrin-3, although it did not reach statistical significance (not shown).

### 3.6. OLE Reduces the Inflammatory Response in Microglial Cells In Vitro

To evaluate whether the protective effects found in vivo in OLE-treated EAE mice involve direct actions on relevant CNS inflammatory cells, we studied the effects of OLE on activated BV-2 microglia cells, in order to mimic responses observed on neuroinflammatory disorders.

**Figure 6:**
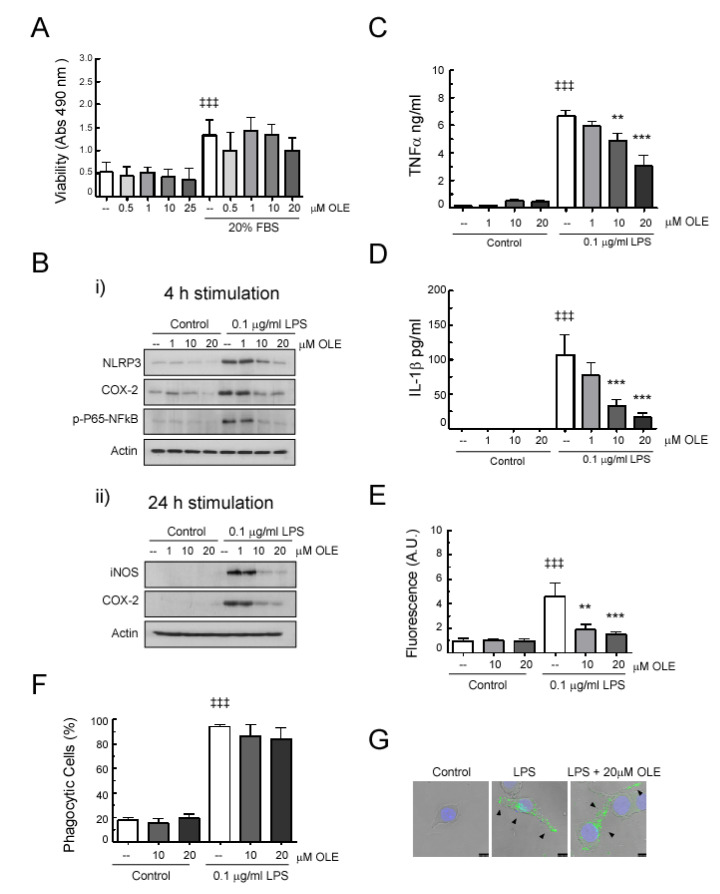
Oleacein treatment modulates BV-2 microglia cell activation. BV-2 cells were pretreated for 30 min with the indicated doses of OLE. (**A**) Cell viability was assayed after 24 h of incubation in the absence or presence of 20% FBS (^‡‡‡^ *p* < 0.001 vs. without FBS; n = 3). (**B**) After 4 h (**i**) or 24 h (**ii**) of stimulation with 0.1 µg/mL of LPS, COX-2, iNOS, phosho-p65-NFκB and NLRP3 expression was identified in cell lysates by Western blot, and (**C**) the presence of TNFα and (**D**) IL-1β in the 24 h cell culture medium was quantified by commercial ELISA (^‡‡‡^ *p* < 0.001 vs. control, *** *p* < 0.001 and ** *p* < 0.01 vs. stimuli without OLE; *n* = 3). (**E**) After 24 h of stimulation with 0.1 µg/mL of LPS, intracellular ROS production was evaluated by Flow cytometry analysis: Bar graph shows quantification expressed in arbitrary units (A.U.) (^‡‡‡^ *p* < 0.001 vs. control; *** *p* < 0.001 and ** *p* < 0.01 vs. stimuli without OLE; *n* = 3). (**F**,**G**) After 24 h of stimulation with 0.1 µg/mL of LPS, cells were incubated with 1 mg/mL of FITC-labelled dextran, and phagocytosis was measured by Flow cytometry analysis: Bar graph shows percentage of BV2 cells expressing phagocytic activity. Representative confocal microphotographs of cells are shown, verifying that green fluorescent (FITC-dextran) associated with BV-2 microglia is localized inside of the cells (arrowheads). ^‡‡‡^ *p* < 0.001 vs. control (*n* = 3).

First, we assessed the potential cytotoxicity of OLE in BV-2 microglia using the MTT colorimetric assay. As shown in Figure 6, OLE did not have any cytotoxic effect on BV2 cells after 24 h, regardless of the dose (1–24 μM) (Figure 6A).

Next, to determine whether OLE was able to directly modulate inflammatory activity on microglia, BV-2 cells were pretreated with 1, 10 and 20 μM of OLE for 30 min and then stimulated with LPS (0.1 µg/mL) for 4 h and 24 h. As shown in Figure 6B and Figure S1, rapid key proinflammatory events, such as phosphorylation of p65-NFκB and overexpression of NLRP3, were observed up-regulated after 4 h of cell stimulation with LPS, and OLE reduced this upregulation. Moreover, cell pretreatment with OLE also led to a dose-dependent inhibition of LPS-induced iNOS and COX-2 production, whereas OLE did not affect their basal expression per se. In consonance, the presence of OLE also reduced the ability of LPS to induce TNFα and mature IL-1β secretion in a dose-dependent manner (Figure 6C,D, respectively). Induction of iNOS expression was not evident after 4 h LPS stimulation (data not shown).

Given that ROS production may shape specific inflammatory programs, we also investigated intracellular ROS accumulation. Flow cytometry analysis showed that intracellular ROS build up, which was significantly increased in LPS-treated BV-2 cells, was dramatically suppressed in cells pretreated with different doses of OLE (Figure 6E).

Finally, we also determined the direct effect of OLE on the phagocytic properties of activated microglia. As shown in Figure 6F,G, the enhanced phagocytic capacity of LPS-stimulated BV-2 cells remained unaltered by preincubation with OLE. Fluorescence of dextran beads was restricted to the interior of the cell, as seen in the confocal images, thus confirming cellular uptake.

### 3.7. OLE Reduces In Vitro the Oxidative Response in Retinal Ganglion Cells

**Figure 7:**
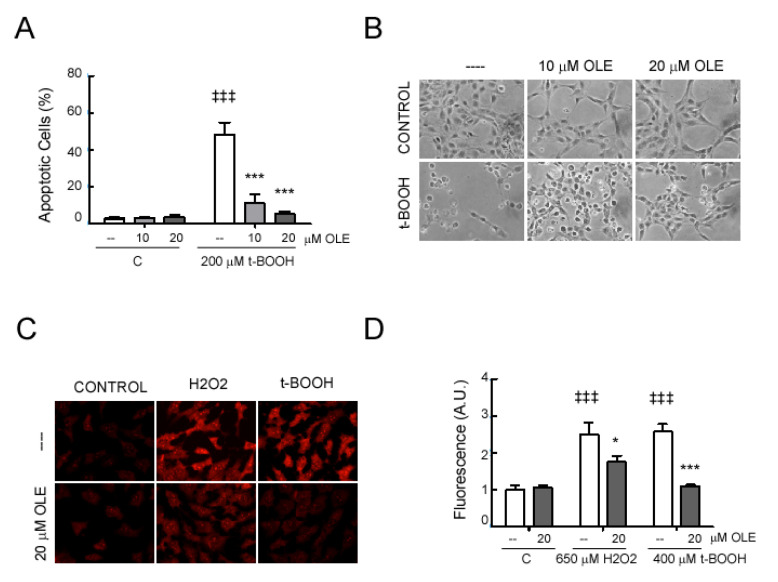
Oleacein treatment modulates RGC-5 activation. RGC-5 cells were pretreated for 30 min with the indicated doses of OLE. (**A**) Cell apoptosis was assayed after 24 h of incubation in the absence or presence of 200 µM of tert-butyl hydroperoxide (t-BOOH). . Apoptotic cells were estimated by Flow cytometry analysis. ^‡‡‡^ *p* < 0.001 vs. control; *** *p* < 0.001 vs. stimuli without OLE; *n* = 3. (**B**) Representative bright-field microscopy images are shown. (**C**,**D**) After 24 h of stimulation with 400 µM of t-BOOH or 650 µM of H_2_O_2_, cells were stained with DHE and analyzed by fluorescence microscopy: Quantification expressed in arbitrary units (A.U.), and representative microphotographs of cells labeled with DHE: ^‡‡‡^ *p* < 0.001 vs. control; *** *p* < 0.001 and * *p* < 0.05 vs. stimuli without OLE; *n* = 3.

To investigate the effect of OLE on retinal ganglion cell death, RGC-5 cells were exposed to the exogenous oxidant tert-butyl hydroperoxide (t-BOOH). As shown in Figure 7A the percentages of apoptotic cells raised to 48.4 ± 6.3% from 2.7 ± 0.7% without stimulus at 24 h (*p* < 0.001). After coincubation with OLE, the apoptotic cells due to t-BOOH treatment, significantly decreased at both 10 and 20 µM (11.5 ± 4.8% and 5.2 ± 1.2%; *p* < 0.001; respectively). Representative microphotographs are shown in Figure 7B.

Since the protection observed could be due to the inhibition of the ROS production by OLE, we checked ROS activity, using the redox-sensitive fluorescent probe DHE, as we previously did in the tissues. In Figure 7C we observe that incubation with OLE reduced the superoxide anion production induced by both H_2_O_2_ and t-BOOH. Quantification of the result is shown in Figure 7D.

## 4. Discussion

In this report, we evaluated the protective effect of treatment with OLE in the mice model of MOG-induced EAE. We observed that OLE administration improved neuromotor disabilities associated with EAE and reduced CNS damage. Therefore, and with the obvious reservations, due to species change, our data present a promising bioactive molecule, derived from a natural source, EVOO, candidate for further research in the treatment of MS and possibly other immune–inflammatory-related diseases.

OLE is a degradation product released from endogenous β-glucosidases acting on phenolic glycosides during the process of olive oil extraction and maturation [18]. In fact, it is one of the major secoiridoid phenolic compounds present in EVOO, the main fat source in the Mediterranean diet [19]. Several studies, either in vitro or in vivo, in experimental animal models and in human immune cells, have demonstrated the potent antioxidant and anti-inflammatory profile of the OLE, which supports its potential role in the prevention and treatment of various pathological conditions [20,21]. Our group and others have shown positive effects of other olive oil derivatives in an in vivo model of MS, but, to our knowledge, this is the first time in which OLE is studied in the context of MS [22,23,24].

The clinical and pathophysiological processes of EAE were confirmed in this study by the remarkable increases in IgG autoantibodies, augmentation in neurologic functional scores and development of inflammatory infiltration and demyelination in CNS tissues, including the spinal cord, cerebellum and optic nerve, of EAE mice. These findings were consistent with earlier reports [22,23].

Our data demonstrated that OLE administration to EAE-mice delayed the motor clinical signs of the disease and lessened pathological damages in the central CNS. The histopathological results indicated that attenuation of EAE pathogenesis by OLE came along with a reduction of both inflammatory cells infiltration and demyelination of the CNS tissues studied (spinal cord, cerebellum and ON), and by prevention of BBB disruption; all of these events are typical pathological hallmarks observed in MS patients. In agreement with our promising results, it has already been demonstrated that OLE also reduced both the infiltration of macrophages and lymphocytes in adipose tissue, in an experimental model of high fat diet (HFD)-induced adiposity and, in the liver, the development of HFD-dependent hepatic steatosis [3,4].

Additional findings revealed that OLE intervened in the disease progression through the inhibition of the synthesis of inflammatory cytokines, such as TNFα, IL-13, IL-33 in spinal cord tissue; concomitantly increasing the production of the anti-inflammatory cytokine IL-10. At systemic levels, the presence of TNFα and IL-1β was also abrogated in OLE-treated EAE mice. High levels of these inflammatory cytokines have been earlier observed in peripheral and CNS of both EAE mice and MS patients, and their deleterious effects include the ability to induce the expression of adhesion molecules, chemokines, disruption of the BBB and, consequently, the recruitment of autoreactive T cells into the CNS [25,26,27,28].

The alarmin IL-33, which primary source in the spinal cord is astrocytes, influences microglial activation and secretion of inflammatory cytokines and chemokines, such as IL-6, IL-13 and MCP-1. It also increases endothelial permeability, which generates BBB alteration and facilitates recruitment of leukocytes into the CNS [26]. Our data showed that IL-33 levels were reduced in spinal cord of EAE mice treated with OLE. Although the precise role of IL-33 in EAE/MS remains to be clarified, our findings agree with a previous report, where levels of IL-33 in the CNS correlate with EAE severity, being also in line with treatments, such as vitamin D or anti-CD52, in which the reduction of IL-33 expression levels in spinal cord tissue is associated with an improvement of both clinical symptoms and neuroinflammation, observed in EAE-treated mice [29,30,31]. Moreover, the treatment of EAE mice with OLE restored the expression levels of IL-10 in spinal cord tissue. In fact, OLE was able to raise the expression of this cytokine in control healthy mice, as a sign of an anti-inflammatory profile triggered by the secoiridoid already found previously [23]. An increase in the immunosuppressive cytokine IL-10 has been associated with an amelioration of EAE-induced disease manifestations following administration of an anti-IL-33 antibody [32]. In fact, some of the classical disease-modifying therapies have an effect in increasing this anti-inflammatory cytokine in MS patients [33,34].

Another interesting finding from our study was that the administration of OLE prevented the increase in the expression of the inflammatory chemokine MCP-1 in the spinal cord of EAE-affected mice. MCP-1 is associated with acute disease symptoms, as well as with initiation of the blood-brain barrier breakdown [35,36]. Descriptive studies of MS have identified that MCP-1 plays a key role at the BBB and parenchyma, encouraging the recruitment of monocytes and T cells from blood [37]. In accordance, concomitant with a diminution of MCP-1 expression we observed in this study a reduced infiltration of inflammatory cells into CNS tissues and a protection from BBB disruption in OLE-treated mice.

We also carried out analysis of GM-CSF, a central mediator of tissue inflammation. As previously reported, we found a significant increase of this cytokine in both spinal cord tissue and blood serum of EAE-untreated mice compared to controls [38]. It has been well documented that GM-CSF, predominantly expressed by autoreactive helper T cells, plays a critical role in promoting the migration of myeloid cells across the BBB, and once in the parenchyma, it supports their differentiation into pathogenic effectors expressing inflammatory mediators including IL-1β, IL-6, TNFα and ROS, which participate in tissue damage during EAE [39,40]. Therefore, the reduced local and systemic GM-CSF expression observed in OLE-treated EAE mice might be responsible for the downward trend in the number of cells infiltrating into the CNS which promote inflammation and demyelination in EAE mice. It should be noted, that the decrease in GM-CSF expressing cells in the CNS of EAE mice has been identified as a component of the immunomodulatory mechanism of action of first-line disease-modifying drugs such as dimethyl fumarate and glatiramer acetate [41,42]. In humans, the immunomodulator dimethyl fumarate decreases GM-CSF-producing Th1 cells in peripheral blood mononuclear cells from healthy subjects. Accordingly, MS patients treated with glatiramer acetate also show low serum levels of GM-CSF [41,42]. Thus, the GM-CSF downregulation observed in this study might also aid to explain the beneficial effect of OLE.

Along with changes in the inflammatory profile triggered by OLE, we observed adjustments in the oxidative stress parameters, which are also associated with the pathogenesis of inflammatory demyelination and other neurodegenerative diseases [43,44,45].

Massive production of ROS is involved in the cascade of events that leads to cell death and neuronal damage in EAE and MS, pointing to activated microglia and macrophages as the major source of these radicals in MS lesions [46,47,48,49]. We observed that the excessive ROS build up in CNS tissues including spinal cord, cerebellum and optic nerve of EAE-mice, remarkably decreased in OLE-treated EAE mice. ROS induce cytoskeletal changes in brain endothelial cells, thereby affecting BBB integrity and facilitating transendothelial monocyte migration, as well as myelin sheath damage [50,51]. These events result in BBB breach and CNS demyelination, as seen in untreated EAE-mice, and in turn, OLE administration prevented all these pathogenic outcomes.

Prominent ROS accumulation in the optic nerve contributes to RGCs apoptosis and axonal loss, being the major cause of visual loss in EAE/MS disease. It has been reported that RGCs, whose axons contribute to the ON, are highly dependent on oxidative phosphorylation and increasing antioxidant defenses against superoxide provides neuroprotection [52]. In our in vitro study, the presence of OLE, clearly diminished both ROS production and, consequently, apoptosis of neuronal cells (RGC-5 cells), supporting the axis “ROS-cell loss”. Further studies are needed to elucidate whether OLE action may protect from loss of RGCs and visual function in MOG-induced optic neuritis, given that both accumulation of oxidative stress and demyelination in the optic tract nerves, were substantially reduced in our mice model under OLE treatment.

The high ROS production observed in EAE-affected mice leads to oxidative damage of cell components, such as proteins and lipids, which may explain the simultaneous elevated levels of lipid hydroperoxides and protein oxidation products detected in both spinal cord and serum of mice with the disease, based on MDA and AOPP determinations, respectively. As expected, OLE treatment resulted in a significant decrease in the elevated MDA and AOPP levels, reflecting a protection from lipid and protein damage; significant increases in FRAP and Sestrin-3 serum levels, as antioxidant defense elements negatively affected by EAE definitely contributed to the reduction of oxidation. Sestrin-3 is an intracellular sensor belonging to a family of proteins that act as antioxidants to reduce intracellular ROS levels and/or its reactive metabolites by regenerating overoxidized peroxiredoxins that, in turn, deoxidize ROS [53,54]. To our knowledge, there is no information available unveiling the role of Sestrin-3 in EAE/MS, but different investigations have determined that sestrin deficiency renders cell tissues to oxidative stress, while, when overexpressed, they are protected from oxidative damage [54,55,56,57].

Furthermore, in this report we found that OLE treatment can be a direct modulator of the potentially damaging responses of microglia. Activated microglia release proteolytic enzymes, cytokines, oxidative products, and free radicals, thus influencing different phases of the MS disease process. By in vitro approaches, OLE was shown to inhibit microglial activation that may contribute to CNS tissue injury. Thereby, OLE pretreatment resulted in abrogation of LPS-induced oxidative response, as well as suppression of the expression of IL-1β, TNFα, COX-2 and iNOS over the time in BV2 microglial cells. The long term-abrogation of this expression prompted us to check on the involvement of transcription factors in the action of OLE. Actually, activation of the transcription factor NFκB and induction of the NLRP3 inflammasome, both key regulators of proinflammatory genes, were also under control in the presence of OLE. A similar mechanism has been found for other natural phenolic compounds with anti-inflammatory properties [58].

However, microglial activities within the CNS are complex, and along with harmful effects, microglia also exert beneficial roles, including clearance of myelin debris [59]. Fortunately, OLE treatment did not affect the phagocytic response induced by LPS. This fact positions OLE as a valuable compound, blocking selectively the unwanted responses, but not limiting the normal cell function. Therefore, OLE has inhibitory effects on the proinflammatory microglia phenotype, without affecting the hallmark of microglia: its ability to phagocytose. Similar effects have already been reported for the drug dimethyl Fumarate, which is the current treatment for Multiple Sclerosis [60].

In summary, there is a correlation between the inflammatory and oxidative status of the CNS and EAE-induced pathological changes in the CNS, and, based on this study, we suggest that OLE may provide protective effects in EAE by reducing leukocyte infiltration into the CNS, preventing inflammatory mediators and inhibiting the elevation of the oxidative stress status.

## Figures and Tables

**Table 1:** Elution systems and fractions for the isolation of oleacein.

Mobile Phase Cyclohexane: EtOAc	Fractions	Volume (mL)
100% cHex	1	500
95 cHex: 5 EtOAc	2–22	550
90 cHex: 10 EtOAc	23–152	2840
88 cHex: 12 EtOAc	153–155	200
85 cHex: 15 EtOAc	156	200
80 cHex: 20 EtOAc	157–284	3850
75 cHex: 25 EtOAc	285–340	2800
50 cHex: 50 EtOAc	341–359	1200
100% EtOAc	360–374	950

Fractions 250–276 (40 mg) contained oleocanthal and fractions 351–359 (65 mg) contained oleacein. The identity of the compounds was confirmed by ^1^H-NMR. The purity was confirmed by qNMR and was >95%.

## Supplementary Materials

The following are available online at https://www.mdpi.com/2076-3921/9/11/1161/s1, Figure S1: Oleacein treatment modulates BV-2 microglial cells activation: Quantification.
