# Supplementary material for: Oleacein Attenuates the Pathogenesis of Experimental Autoimmune Encephalomyelitis through Both Antioxidant and Anti-Inflammatory Effects
Source: Antioxidants (Basel). 2020 Nov 21;9(11):1161. doi: 10.3390/antiox9111161 (PMC7700216; doi:10.3390/antiox9111161)
Supplement: Supplementary file 1 [file antioxidants-09-01161-s001.pdf]

Legend of Figure

Figure S1. Oleacein treatment modulates BV-2 microglial cells activation: Quantification. BV-2 cells were pretreated for 30 min with the indicated doses of OLE. After 4 (i) or 24 (ii) h of stimulation with 0.1 µg/ml of LPS, COX-2, iNOS, phosho-p65-NFκB and NLRP3 expression was identified in cell lysates by Western blot. Quantification graphs of the Western blots are shown with fold over the control in each condition (‡‡‡p<0.001 *vs* control; ***p<0.001, **p<0.01 and *p<0.05 *vs* stimuli without OLE; n= 3)

Figure S1

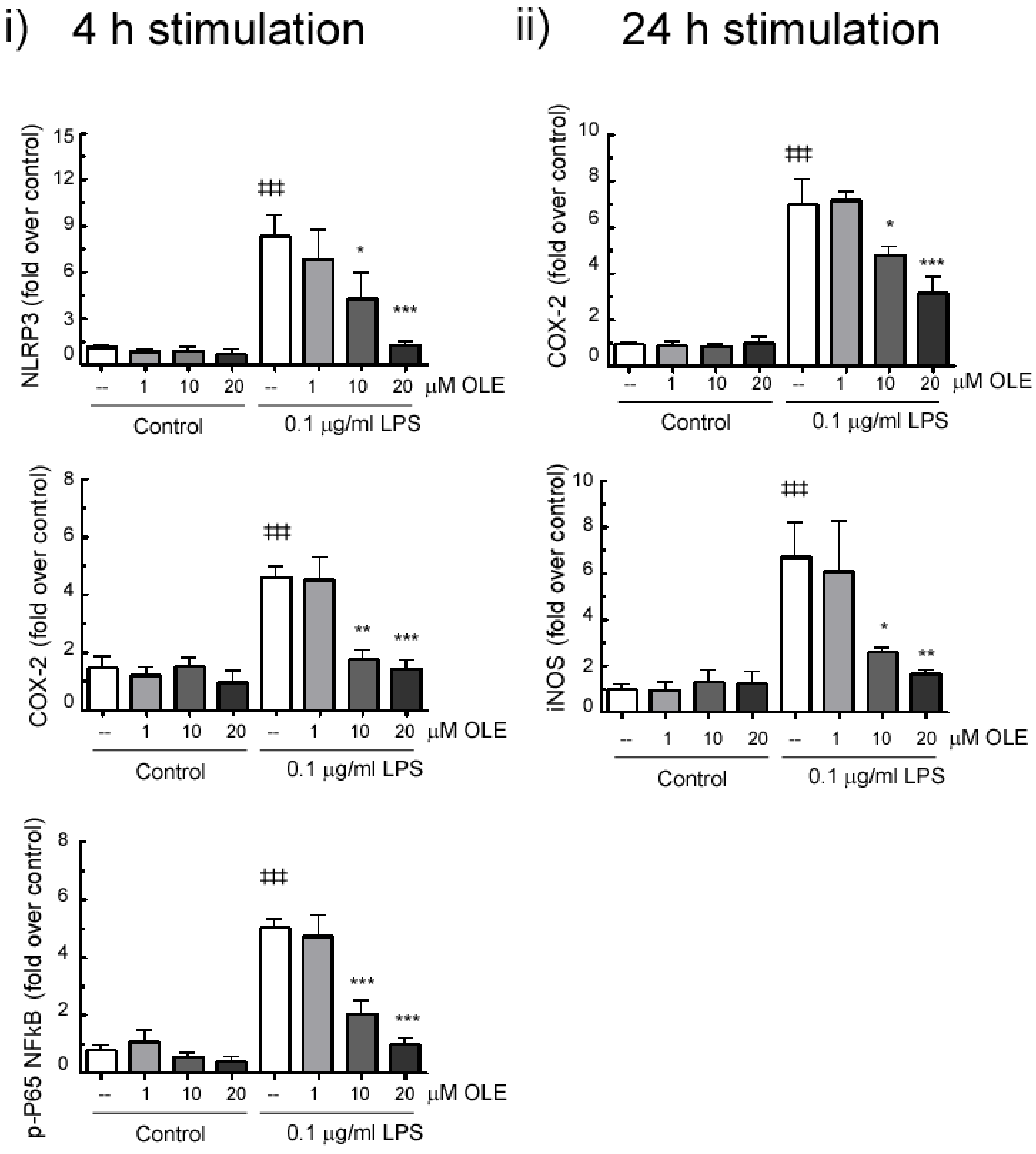

i) 4 h stimulation
ii) 24 h stimulation
NLRP3 (fold over control)
COX-2 (fold over control)
p-P65 NFkB (fold over control)
iNOS (fold over control)
‡‡‡
*
**
***
-- 1 10 20 -- 1 10 20 μM OLE
Control
0.1 μg/ml LPS
